# Modelling human musculoskeletal functional movements using ultrasound imaging

**DOI:** 10.1186/1471-2342-10-9

**Published:** 2010-05-21

**Authors:** Michael Peolsson, Tommy Löfstedt, Susanna Vogt, Hans Stenlund, Anton Arndt, Johan Trygg

**Affiliations:** 1 Computational Life Science Cluster (CLiC), Umeå University, Umeå, Sweden; 2 Department of Chemistry, Umeå University, Umeå, Sweden; 3 School of Technology and Health, Royal Institute of Technology, Stockholm, Sweden; 4 Department of Clinical Science, Intervention and Technology, Division of Orthopeadic Surgery, Karolinska University Hospital, Huddinge, Stockholm, Sweden; 5 Swedish School of Sport and Health Sciences (GIH), Stockholm, Sweden

## Abstract

**Background:**

A widespread and fundamental assumption in the health sciences is that muscle functions are related to a wide variety of conditions, for example pain, ischemic and neurological disorder, exercise and injury. It is therefore highly desirable to study musculoskeletal contributions in clinical applications such as the treatment of muscle injuries, post-surgery evaluations, monitoring of progressive degeneration in neuromuscular disorders, and so on.

The spatial image resolution in ultrasound systems has improved tremendously in the last few years and nowadays provides detailed information about tissue characteristics. It is now possible to study skeletal muscles in real-time during activity.

**Methods:**

The ultrasound images are transformed to be congruent and are effectively compressed and stacked in order to be analysed with multivariate techniques. The method is applied to a relevant clinical orthopaedic research field, namely to describe the dynamics in the Achilles tendon and the calf during real-time movements.

**Results:**

This study introduces a novel method to medical applications that can be used to examine ultrasound image sequences and to detect, visualise and quantify skeletal muscle dynamics and functions.

**Conclusions:**

This new objective method is a powerful tool to use when visualising tissue activity and dynamics of musculoskeletal ultrasound registrations.

## Background

Image analysis has been used for decades as a tool to analyse different kinds of tissues. It provides a rich resource when presenting medical and clinical information and the plethora of techniques using images is increasing rapidly.

Various pathologic conditions benefit from medical imaging of soft tissues in terms of detection, progression, remission (effects of treatment), etc. Tumours, for example, share important similarities with skeletal muscles. They are both inherently smooth masses with dynamic characteristics. In the tumour case, a change in the tissue's dynamic quality is an important indicator of diagnostic evaluations and is therefore of great clinical value. For instance, decreased tissue dynamics in a muscle can, in the musculoskeletal case, be an indication of inflammation [1] or ischemia [2]. Muscle function can also be related to a wide variety of conditions, for example pain, ischemic and neurological disorders, exercise and injury. There is thus an urging need for imaging tools that can be used to study the dynamics of skeletal muscles, and especially to do so in real-time.

The quality of ultrasound imaging has evolved quickly and is today capable of producing high spatial resolution image sequences with a high frame rate. This makes ultrasound imaging suitable for analysis of both structural and functional aspects of muscles. Examples include tendon injuries in sports medicine [3] and inflammation processes [4,5].

Speckle tracking is a technique that can be applied to ultrasound sequences. Speckle tracking relies on the scattered echoes that arise from inhomogeneities in the tissues. Speckle tracking provides studying tissue motions in real-time by following a specific pattern frame-by-frame in a sequence of images [6]. The speckle tracking can be described in terms of e.g. displacement maps [7-11]. Our studies here are focused mainly on transverse displacements, but are performing 2D measurement; a recent study has shown that measurements of 2D displacements can be accurately performed [12]. Skeletal muscle dynamics and muscle fatigue have also been studied by using speckle tracking [13,14].

The aim of our study is to develop a complementary method that can be used separately for analysis of skeletal muscles, or used together with current and established methods—such as speckle tracking—to improve model precision. The method that we present here is based on two parts: the discrete wavelet transform and multivariate data analysis. The wavelet transform is able to extract position, size and shape information that is present in greyscale B-scan ultrasound image sequences of muscle tissues movements. Not only are single pixel intensities considered, but regions of pixel intensities are considered and extracted as a whole. Secondly, multivariate analysis is applied to the wavelet transformed images in order to get a global model of the captured events. Multivariate analysis allows a multitude of different tools to be used on the data, e.g. comparisons, cluster analysis, discrimination analysis, etc; and helps to detect, visualise and quantify the skeletal muscle dynamics and functionality. Multivariate methods can also analyse speckle tracking data, as we will demonstrate in the given examples.

The intensity value changes within an image represent movements directly observable (the effect of noise is heavily reduced by using the MACI method) and which can for example, in the speckle tracking case, be directly and actively followed, "observed", around within an image in an image sequence. Using the discrete wavelet transform means instead that these intensity value changes, which represent actual muscle tissue movements, are indirectly and passively observed as they *pass by *the "observer" (the variable, the wavelet coefficient), meaning a particular muscle tissue movement is not represented by any single variable, but by a multitude of variables simultaneously and together describing a particular phenomenon within the sequence.

The MACI method is applied to describe the dynamics in the Achilles tendon and the calf during real-time heel raise movements. It will be argued that this technique is a valuable tool to use when studying and visualising muscle tissue dynamics.

The strategic significance of a method like MACI is very high since it could be applied to muscle rehabilitation programmes (including sports medicine), neurological disorders (e.g. whiplash and fibromyalgia) and pain related conditions (e.g. back pain).

### Modelling tissue dynamics

The basis for image analysis is that an *M *× *N *two-dimensional digital intensity image can be seen as a function, *I*(*x, y*), with an intensity value for every point, (*x, y*), of the image [15].

When several such 2D images are stacked upon each other, like in an ultrasound image sequence, a multivariate image space occurs. In such a multivariate image space, each location is represented by a multitude of values and a massive amount of highly correlated data is the result. Different unfolding procedures can then be used in order to analyse these 3D data structures [16-18].

The feature extraction procedure used here is based on the discrete 2D wavelet transform (2D-DWT). The 2D-DWT is beneficial to use here since when using it to transform and compress large sets of images, *the important position, size and shape information in the images is not lost*. The images are compressed by choosing the wavelet coefficients that hold *the most *information about the ultrasound sequence. This is done by measuring the variance of each coefficient. The selected wavelet coefficients are then put in a regular two-way data table where each row represents an image and each column contains the wavelet coefficients for that image. Principal component analysis (PCA) is then used to analyse the two-way data table of wavelet coefficients.

In the examples below, movements in the Achilles tendon and the calf were analysed by multivariate analysis of congruent images (MACI). The results were then compared to an analysis of the same movements in the corresponding areas using state of the art speckle tracking. Specifically, the absolute transversal displacement was measured using speckle tracking and the results were compared to the results of the MACI method. Also, a grid of square speckle tracking ROIs was placed equidistantly in the whole image plane in the third example and a PCA of the resulting tracks was made. The purpose of using these speckle tracking measures was to show that the MACI technique indeed does capture the actual movements in the sequence.

The investigated types of movements are: standardised passive movement, standardised active movement and functional movement.

## Methods

The MACI method was used in the example cases below to extract information from ultrasound image sequences. The resulting two-way data table was examined with PCA and the movements verified by speckle tracking. This section explains speckle tracking, PCA and the concept of MACI.

### Speckle tracking

The acoustic patterns in an ultrasound signal change when the muscle being scanned moves. These acoustic markers, or speckle patterns, remain relatively stable over time and can therefore be followed frame-by-frame in a sequence of images [19]. A commercial software package (EchoPac, GE Healthcare, Horten, Norway) was used together with the ultrasonic system Vivid 7 (GE Healthcare, Horten, Norway) in all case examples in this study. An in-house developed speckle tracking software was also used in the third example.

The first step in speckle tracking is to specify a rectangular region of interest (ROI) in a particular frame. The method finds the corresponding region in the next frame that is most similar to the selected region, by some criterion. The objective is thus to find the values of Δ*x *and Δ*y *that minimise

Where *I *is the image intensity, *x *and *y *are the pixel coordinates at time, or frame, *t *and *w *is a weighting function, which can be 1 in the simplest case.

The speckles are not always followed correctly when there are rapid alterations in the tissue, when there are large deformations in the tissue or when there are out of plane motions present. However, current research indicates that speckle tracking indeed is well suited for skeletal muscle investigations [20,21].

The speckle tracking algorithm used in the in-house developed speckle tracking software is the Lucas-Kanade optical flow method [22] using pyramids [23] (hierarchical version) as implemented in the open source computer vision library (OpenCV) version 1.1 http://opencv.willowgarage.com/wiki/.

Some more information regarding greyscale B-scan ultrasound and speckle tracking is given in the supplemental material in Additional file 1.

### Principal component analysis

Principal component analysis (PCA) is commonly used to identify patterns in multi- and megavariate data sets [24].

PCA is one of the cornerstones of multivariate data analysis. It is used to extract systematic variation in a dataset by projecting the data to a lower-dimensional space in which they are more easily analysed. PCA achieves this by finding the best least squares approximating hyperplane in this lower-dimensional space.

PCA can be computed by iterative methods, such as nonlinear iterative partial least squares (NIPALS), or for example by eigenvalue decomposition. When PCA is done, three matrices will constitute the result and they are related by

where *k *is the number of latent variables, **T **contains the scores, **P **contains the loadings and **E **is a matrix of residuals. **X **is the matrix of data being analysed.

The scores give information about the relation between the observations, for example the variation between the frames of an ultrasound sequence. Plotting score vectors against each other results in a *score plot *that gives information about trends, clusters and outliers in the data.

The loadings are seen as a measure of the importance of the variables. The loadings can be used to reconstruct the ultrasound sequence, resulting in a *loading image*. This image gives information about what parts of the frames are important for describing the movement.

PCA was performed using SIMCA-P+ 11.5 (Umetrics, Umeå, Sweden).

### Multivariate analysis of congruent images

Multivariate analysis of congruent images (MACI) is used to find and express patterns over multivariate image spaces for the purpose of classification of, or finding relationships between, the images [25]. The main goals of multivariate image analysis are: firstly, to compress the highly correlated data into terms of a few linear combinations of the intensity values; and secondly, to preserve the spatial information in the images.

By *congruent *is meant a set of images that are properly pre-processed, i.e. transformed, so that each image element corresponds to the same element across all images. When the images are not fully congruent initially, as they seldom are, they are made so by some means. In this case the discrete 2D wavelet transform (2D-DWT) of the images was used to make them congruent. The 2D-DWT was used to extract spatial (position) and frequency (shape and size) based features from the images. The wavelet basis Symlet 8 was used in the transforms.

The wavelet coefficients for each image are put into the rows of an ordinary two-way data table on which conventional multivariate methods, such as PCA, can be used to extract the information. In such methods, each variable must describe the same phenomenon for each observation; this is solved by using the congruent wavelet coefficients.

The development of the wavelet texture analysis-based methods have, in recent years, opened the door to this new field of multivariate data analysis [26].

**Figure 1:**
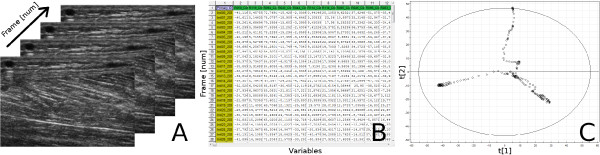
**The schematic principle of MACI**. The principle of transforming an ultrasound image sequence (a) to PCA (c) by means of the wavelet transform (b). A multivariate image space, an ultrasound loop—a series of images. (b) The congruent images are put as the rows of an ordinary two-way data table. This means that each row corresponds to a point, or frame, in time. The columns contain wavelet coefficients. (c) A two-component PCA score plot of the table of wavelet coefficients, i.e. the second score vector, **t2**, plotted against the first score vector, **t1**.

To summarise the principles of MACI, an overview of transforming B-scan ultrasound images to a PCA score plot by using the 2D-DWT is presented in Figure 1.

### Experimental setup

Three case examples will be described in this manuscript, and they will be used to illustrate the usefulness of our method. The first example is of a standardised passive ankle movement; the second of a standardised active ankle movement; and the third of a functional ankle movement. The reason for these choices of case examples is that treatment and rehabilitation of patients with ruptured Achilles tendon surgery covers the described chosen movement types in the post-surgery rehabilitation phase.

The movement examined in the examples is dorsal-plantar flexion. The reason for this choice of movement is that it can be standardised by using an isokinetic dynamometer (Isomed 2000, D&R Ferstl GmbH, Henau, Germany). The isokinetic dynamometer controlled the muscular activity (EMG and force) and the range of motion (ROM) of the ankle. The EMG and dynamometer could, for instance, confirm that there was no active muscle contribution present in the passive ankle movement.

The passive dynamic and active isometric movements were performed using the isokinetic dynamometer, with the subject laying face down with the ankle fixated onto the dynamometer platform. The functional movement was performed with the subject in a standing position, holding a hand towards the wall in order to balance the body. Two heel raises were performed from this position using one leg at the time.

**Figure 2:**
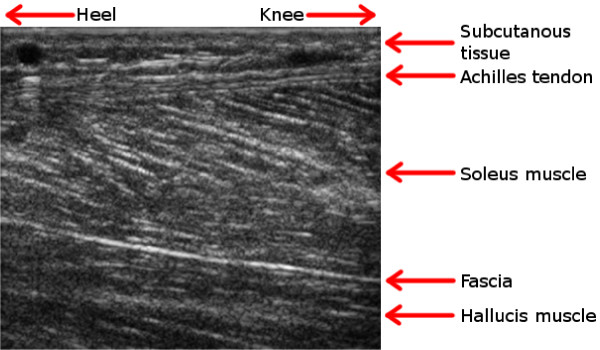
**An ultrasound image**. The anatomic regions in the area around the Achilles tendon are explained by this example ultrasound image. All ultrasound images that occur in this article are captured at about the same anatomic region and are similar to this image. Case Example 1 and Case Example 2 are using a subset of such an image, covering only the Achilles tendon. Case Example 3 exploits the full image.

When capturing the ultrasound loops, the ultrasound system (Vivid 7, GE Healthcare, Horten, Norway) was used together with a linear 12 MHz ultrasound probe. The probe was hand-held and placed in a longitudinal direction over the posterior portion of the Achilles tendon, 3 cm cranially from the lateral malleolus. The anatomic regions that were captured are seen in Figure 2. Movements were captured at 78.6 FPS with a resulting time resolution of approximately 13 ms between frames; the lateral resolution was 0.5 mm.

A commercial speckle tracking post-process software package from GE Medical (Echopac, GE Healthcare, Horten, Norway) was applied to the ultrasound loops in order to provide a reference analysis of the different phases of the ankle movements. An ROI was specified in a vertical position in the tendon, 2 cm proximal to the malleolus. The ROI was specified in the first frame and the speckle tracking method was applied in order to capture the tendon movements in the following frames in a frame-by-frame approach. The ROI was seen to move in accordance with the tissue during the movements. The absolute transversal displacement of the movements was measured, the distance (mm) that each segment of the ROI moved. Information regarding how to interpret the speckle tracking displacement plots is given in the supplemental material, in Additional file 1.

Rectangular ROIs were also specified equidistantly in a 100 × 100 grid over the entire image plane in the third example. This was done using an in-house developed speckle tracking software. The reason for this is to provide a more similar reference to the whole-image analysis that the MACI method performs on the image sequence in the third example.

EMG was used in the active foot ankle movement to confirm baseline rest (not shown).

#### Case study 1: Passive foot ankle movement

The objective here was to analyse the tissue response during a non-active dorsal-plantar flexion movement. This movement is the first type of movement in the clinical rehabilitation phase.

The dorsal-plantar flexions were provided by fixating the foot onto a platform that was connected to an isokinetic dynamometer. The ankle angle was varied at a constant speed of 30 degrees per second between 20 degrees of dorsal flexion and 20 degrees of plantar flexion, forming the range of motion (ROM).

A second objective was to study the repeatability of the method when repeating the dorsal-plantar flexions; therefore two subsequent ROMs were performed.

**Figure 3:**
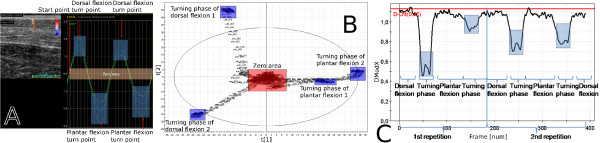
**The results of case example 1**. The ultrasound loop is modelled using the speckle tracking method (a) and the MACI method (b) and (c). Figure (a) illustrates the transversal displacement in the Achilles tendon of two subsequent passive dorsal flexion movements, Figure (b) illustrates the corresponding result of the same movement as a two-component PCA score plot (the second score vector, t2, against the first score vector, t1) and Figure (c) presents the result as distance to model (DModX) plotted against the frame number. Each turning point of the reference movement (a) is found in the corresponding frames at turning points in the PCA score plot (b) and as the best fitting observations in the DModX plot (c).

A 5 × 10 mm speckle tracking ROI was placed in the first frame in the centre of the image field across the Achilles tendon, as seen in Figure 3(a).

#### Case study 2: Active foot ankle movement

The objective in this case was to analyse a movement where the subject activates the calf muscles voluntarily. In order to do so, a standardised active muscle contraction was performed. The reason for this choice was that voluntary muscle activation is the second phase in the rehabilitation program after surgery. In this case, the subject was asked to perform a maximum plantar flexion towards the dynamometer platform (maximum voluntary contraction, MVC). The foot angle in this test was set to 0 degrees.

**Figure 4:**
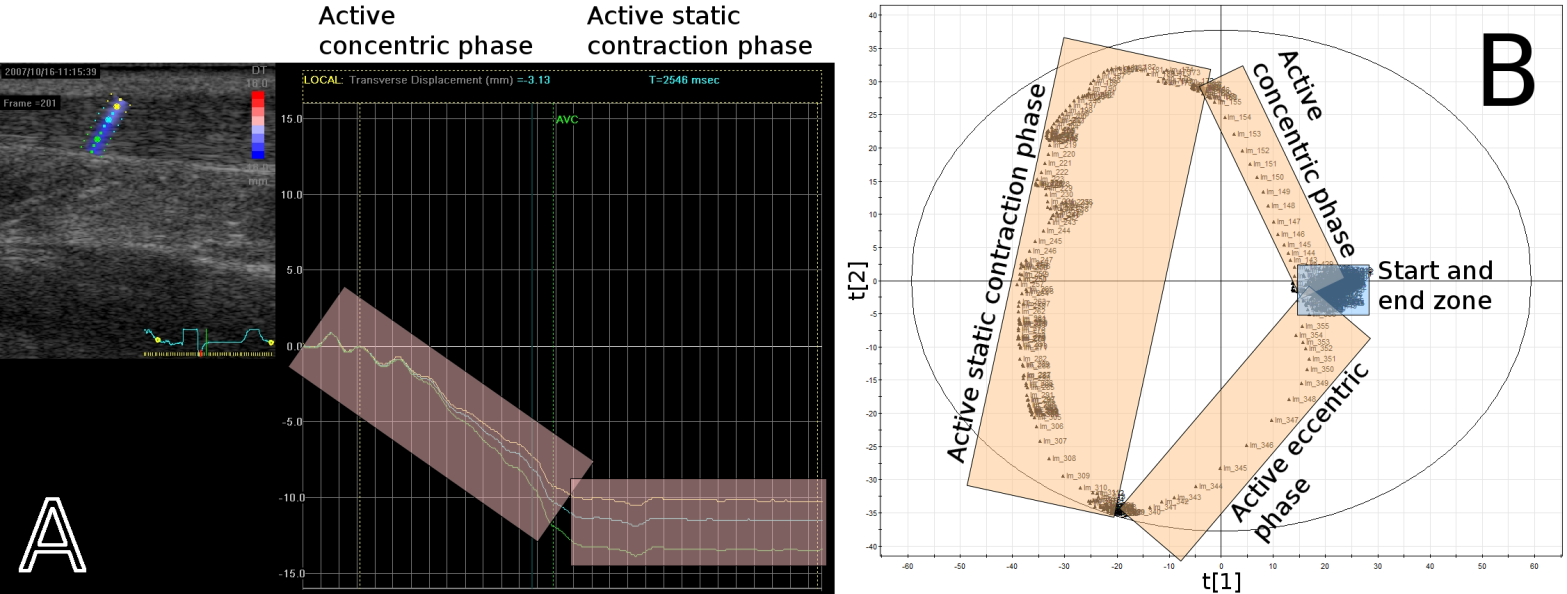
**The results of case example 2.** (a) The plantar flexion movement starts at rest and enters the active concentric phase, which is transformed into a stationary phase, the active static contraction phase, and ends with the active eccentric phase, returning to rest. The last phase is missing in the figure because of a time limitation in the commercial ultrasound software. The eccentric phase is, however, confirmed if the contraction phase is cropped and only the stationary and eccentric phases are examined, or equivalently if the in-house developed speckle tracking software is used. The PCA score plot (b), where the second score vector, t2, is plotted against the first score vector, t1, shows that the start and end zones of the movement are located in the same area of the plot, implying a relaxed muscle before and after the MVC.

A 5×10 mm speckle tracking ROI was placed in the first frame in the centre of the image field across the Achilles tendon, as seen in Figure 4(a).

#### Case study 3: functional movement

The third type of ankle movement studied was a functional movement, in which the ankle was not fixated onto an isokinetic apparatus. The reason for this choice was that functional movements are central in clinical rehabilitation programs in order to regain functionality and smoothness in movements and to prevent secondary lesions. Stabilisation training, by way of balancing, strength training and relearning to use injured muscle structures, is important in rehabilitation.

In this example, the subject performed three subsequent 30 degrees heel raises (plantar flexions) in a standing position. The reason for the choice of three repetitions was to see if it was possible to get an indication of the methodological repeatability when analysing the ultrasound images using the two image analysis techniques.

**Figure 5:**
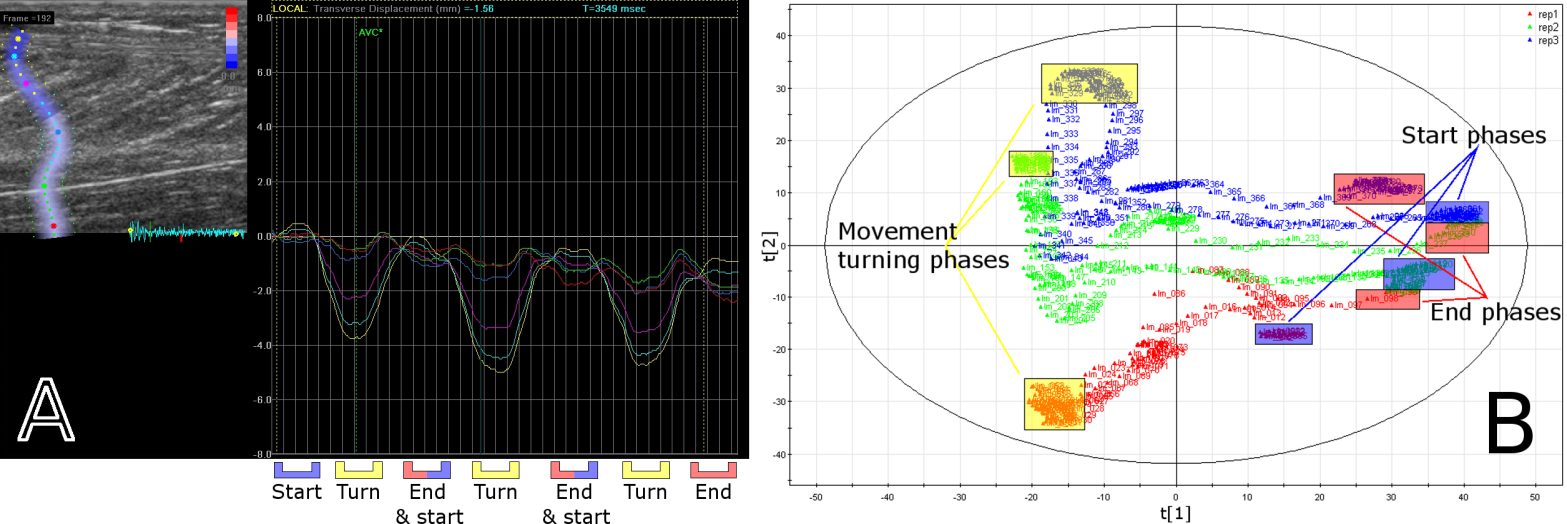
**The results of case example 3**. The similarity of three subsequent heel raises has been analysed. The three repetitions are clearly seen in (a), where they are presented as absolute transversal displacements. A PCA score plot, with the second score vector, t2, plotted against the first score vector, t1, is presented in (b). The speckle tracking results of (a) are found as end or turning points of the trajectories in the PCA score plot (b).

A 5×40 mm speckle tracking ROI was placed in the first frame in the centre of the image field across the whole field, as seen in Figure 5(a).

A speckle tracking measurement of the pennation angle in the Soleus muscle during the movement was performed in order to provide a measurement of the muscle deformation. The pennation angle was measured as the angle of fascicular insertion into the deep fascia of the Achilles tendon. The pennation angles were measured using the in-house developed software and each measure was found as the angle between three points. The pennation angle measurements were confirmed using visual confirmation [27,28].

### Ethical consideration

All experiments were performed on one of the authors of this manuscript. A copy of the written consent of this author for the publication of this case report and any accompanying images is available for review by the Editor-in-Chief of this journal.

## Results

### Case study 1: Passive foot ankle movement

The output from the commercial ultrasound software is shown in Figure 3(a). The original greyscale loop is presented in the upper left part of the image and an ROI is specified vertically within the Achilles tendon. The right part of this and subsequent such images presents the displacement as a function of time for each of the coloured segments of the ROI.

Figure 3(a) presents the speckle tracking result from the tissue movements during two subsequent dorsal flexions. The turning phases of the dorsal and plantar movements are clearly visible.

In Figure 3(b) and Figure 3(c) the MACI result is presented for a sub image covering the Achilles tendon of the same image sequence as in Figure 3(a). PCA was performed on the wavelet compressed images and the result is presented as a score plot and as a distance to model plot, DModX—which is equal to the residual standard deviation [29]. The score plot for this MACI model is presented in Figure 3(b) and the DModX plot in Figure 3(c). The movement phases are highlighted in Figure 3(a) and the corresponding ones in Figure 3(b) and Figure 3(c).

The score plot in Figure 3(b) shows the relationship between the frames of the loop. The score plot shows that each movement starts and ends near to the origin. The first principal component separates the plantar flexions from the dorsal flexions and the second principal component indicates a difference between the two dorsal flexions. The difference between the dorsal flexions is probably due to a slightly unrelaxed muscle in these phases of the movement. The turning phases are easily identified since the observations, the frames, change direction in the score plot.

The DModX plot, Figure 3(c), shows how well different frames were modelled by PCA. Each movement phase and turning zone that was identified in the speckle tracking result can also be identified and correlated in the DModX plot, by simply comparing the frame numbers; the identified frame numbers are found in Table 1. From the DModX plot it is apparent that the model mainly focused on explaining the turning phases—the phases with highest variability—since they have the lowest distance to the model.

**Table 1:** Critical points in the case example movements

	Critical Point	Speckle Tracking	MACI
**Example 1**	Turn, dorsal flexion 1:	56 (700)	57 (712)
	
	Turn, plantar flexion 1:	153 (1934)	152 (1921)
	
	Turn, dorsal flexion 2:	246 (3117)	246 (3117)
	
	Turn, plantar flexion 2:	346 (4389)	345 (4377)

**Example 2**	Concentric phase:	1-156 (0-1972)	1-164 (0-2074)
	
	Static contraction phase:	157-338 (1985-4288)	165-329 (2087-4173)
	
	Eccentric phase:	339-439 (4300-5573)	330-470 (4186-5967)

**Example 3**	Turn, repetition 1:	49 (611)	49 (611)
	
	Turn, repetition 2:	178 (2252)	184 (2328)
	
	Turn, repetition 3:	309 (3919)	312 (3957)

This table provides a means for comparing the time of change of each movement, and each movement phase, for each method. The time of changes was found from the results in Figure 3, Figure 4 and Figure 5.

The frames at which the dorsal and plantar flexions are peaking are presented for Case Example 1. The duration of the movement phases are presented for Case Example 2. The frames at which the plantar flexions are peaking are presented for Case Example 3. The corresponding times in milliseconds are also stated within parentheses. All numbers are approximates and were found from the end of the range of motion for Example 1 and Example 3, and the beginning and the end of the MVC for Example 2.

By comparing the columns of the two methods, it is seen that both methods give very similar results.

Overall, the sequence was modelled well—the two score vectors, created from an initial set of 3000 variables, account for 13% of all variation in the compressed sequence and the observations are within the 5% level in DModX—and all movement phases can be identified and correlated. Similar movement pattern appears in the two subsequent repetitions, both when using speckle tracking and when using the MACI method.

The Euclidean distance between adjacent pairs of images in the PCA score plot in Figure 3(b) reveals important information. Images in the start and end zones and in the turning phase areas are tethered, implying that they are similar. Between the start and end zones and the turning phase areas there are larger distances between the images, implying greater differences. Thus, larger differences are seen where the muscle tissue is either compressed or stretched.

### Case study 2: Active foot ankle movement

Figure 4(a) presents the speckle tracking reference analysis result of the movement, the displacement as a function of time for each of the coloured segments of the ROI. The first two phases—active concentric phase (isokinetic phase), active static contraction (isometric phase)—are clearly visible in the displacement plot. The last phase, the active eccentric phase (isokinetic phase), cannot be plotted together with the two first phases because of a time limitation in the commercial ultrasound software, but the displacement plot does return close to the baseline after the last phase of the movement. This is confirmed if the contraction phase is cropped and only the stationary and eccentric phases are examined, or if the in-house developed speckle tracking software is used.

The MACI result of this case study is presented as a PCA score plot in Figure 4(b). The MACI method was applied to a sub image of the same sequence, a sub image covering the Achilles tendon, just like in the previous case example. All three phases can be identified in the score plot and correlated to the displacement result from speckle tracking, as is also seen in Table 1. This PCA model managed to model the movement very well; the two score vectors, created from an initial set of 3000 variables, that was presented here account for 28% of all variation in the compressed sequence.

The differences in the Euclidian distances between the phases in Figure 4(b) indicate a difference between two kinds of contractions: dynamic isokinetic and static isometric, respectively. In the isometric MVC phase, in the second PCA component direction, it can be seen that clusters of images are separated by images farther apart. Hence, the tissue portrayed in these images both contains images showing hardly any variation (the clusters) and images that are separated by some distance, which suggests dynamic contributions in the tissue in the isometric phase as well. This is a likely result because of the difficulty of maintaining a constant force during the MVC, and MACI thus actually confirms research already made by for example [30]. The distances between the images are larger in the dynamic isokinetic phases, implying more variation, which indicates more dynamics in the tissue during these phases.

### Case study 3: Functional movement

The results of the functional movement analysis are presented in Figure 5. In this case, the speckle tracking ROI was placed in the vertical direction crossing all tissue layers present in the image, instead of just within the Achilles tendon as in the two previous case examples. The right part of Figure 5(a) shows clearly a rhythmic pattern when studying direction, magnitude and internal relationships between investigated segments in the calf.

A rhythmic pattern can also be seen in the PCA score plot in Figure 5(b) for the three sequential movements. All repetitions start in the right part of the score plot and move to the left, they turn and return to the right, close to the start area. The start zone, plantar flexion, turning zone, dorsal flexion and end zones can all be identified and correlated to the movements identified by the speckle tracking method. This can be seen in Figure 5(b) and in Table 1.

The first PCA component in Figure 5(b) separates the plantar flexions from the dorsal flexions. The second component describes inter-repetition variation and the isokinetic turning phase of the plantar flexions—we will return to this in a moment. Thus, the MACI method provides information about both progression characteristics and different phases of the movements as well as information about variations between the three repetitions. It is worth noting that each component carries unique information about the movements and could thus be investigated further.

A comparison of the three repetitions reveals a strong resemblance between them. The PCA scores corresponding to the first and the second repetition have a correlation of 0.64, the first and the third repetition a correlation of 0.72 and the second and third repetition have a correlation of 0.90. The first repetition has a slightly shorter duration than the other two, giving rise to the lower correlation with them.

**Figure 6:**
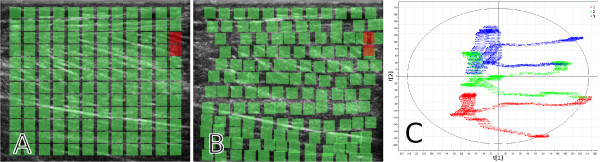
**A whole-image speckle tracking example**. A rectangular grid of equidistantly placed ROIs was specified over the whole image plane, as seen in (a). There is a 12 × 12 grid in (a) for clarity, but in the analysis a 100 × 100 grid was used. (b) illustrates a frame at a later point in time; it is clear that the ROIs have repositioned due to the tissue movements. The red ROIs in (a) and (b) are positions that were tracked badly, in this case they went outside of the right border of the image. The PCA score plot in (c) is found from the two-way matrix created by putting the coordinates of the ROIs as variables in the columns and the time (the frames) in the rows. Note the close resemblance to Figure 5(b).

The second and somewhat different speckle tracking analysis that was made for this case example was performed in order to show that the MACI method indeed does find the same movement phases that are found using speckle tracking. A grid of equidistantly spaced ROIs was specified within the whole image plane, as suggested by Figure 6(a). The ROIs track the underlying tissue over time, see Figure 6(b), and a PCA was performed on the tracked coordinates; the result is presented in Figure 6(c).

It is clear that Figure 6(c) is very similar to Figure 5(b); the two methods have thus captured the same properties of the movements. The first score vector for the three repetitions have almost the same correlation between the repetitions as the MACI method had. However, for the second component, one can see that the start and the end zones of each repetition are much farther apart when using the speckle tracking method than when using the MACI method; something that it is reasonable to believe that they shouldn't be. The reason for this could be due to drift in the speckle tracking, or that the MACI method is better at capturing the isokinetic movement phase found in the second component in the left part of Figure 5(b), before and after the plantar flexion turn.

**Figure 7:**
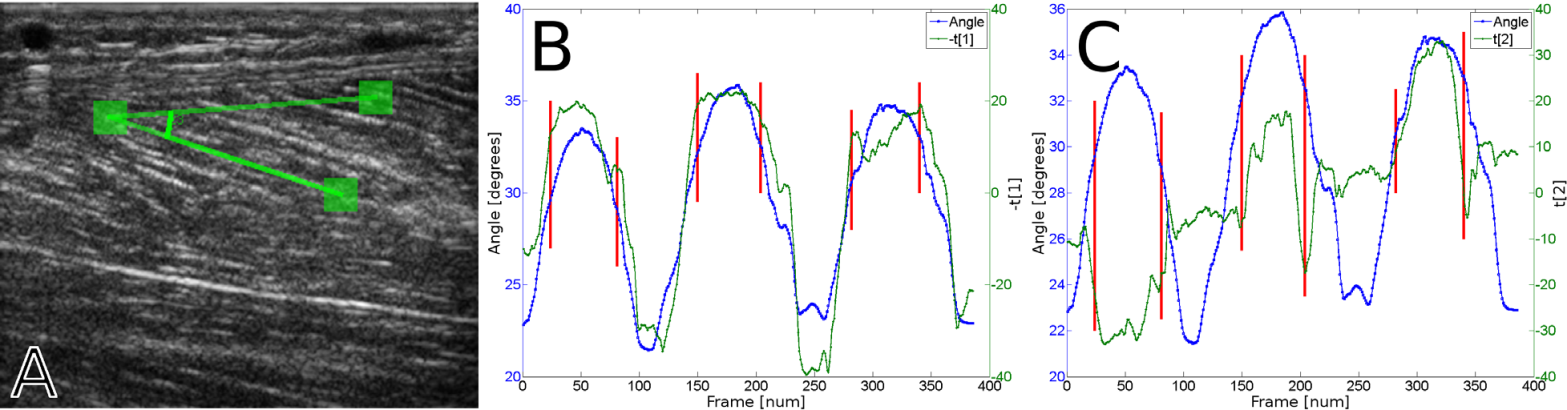
**A muscle deformation example**. The pennation angle in the Soleus muscle was measured over time by using speckle tracking, as illustrated in (a). A representative pennation angle measurement is plotted together with the (negated) first PCA score vector in (b). Note how similar the score vector and the pennation angle are; this means that the muscle deformation that is measured as pennation angle changes in the image sequence is also captured by the first score vector. The second score vector is plotted together with the representative pennation angle in (c). It can be seen that the isometric phase of the movements begins and ends when there are swift changes in the second score vector and only small changes in the first score vector. The isometric phase is thus mainly captured by the second score vector.

The movement being performed contains three elements, or phases, present in all three repetitions: the first is a concentric activation of the Soleus muscle in the plantar flexion in which the area in the images of the Soleus muscle increases (the muscle fibres are contracted). The second phase is an isometric phase containing the movement's turns, in which the area in the images of the Soleus muscle is almost constant. The last phase is an eccentric activation of Soleus in the dorsal flexion phase, returning to rest, in which the area in the images of the Soleus muscle decreases again when the muscle fibres are elongating. The pennation angle was measured as the angle between three points, as suggested by Figure 7(a), in order to provide a measurement of the muscle deformation. A representative speckle tracking measurement of the pennation angle in the Soleus muscle during the movement is presented in Figure 7(b) and Figure 7(c), together with the first and second PCA score vectors.

The frames at which the isometric phase begins and ends are visible in the pennation angle plot, Figure 7(b) and Figure 7(c), as frames at which the angle derivative is slightly smaller. The start and end frames of the isometric phase have been marked in Figure 7(b) and Figure 7(c) for the three repetitions. When the pennation angle is plotted together with the first and second principal component it is clear that the concentric and eccentric contraction phases are mainly caught by the first component and that the isokinetic phases are mainly caught by the second component. This is particularly apparent in the combined two-component score plot in Figure 5(b), where the isokinetic phase corresponds to the left-most, upwards-downwards moving phases of the score trajectory.

## Discussion

Ultrasound images of muscles and tendons captured in real-time during activity carry information about tissue movements and deformations in several different parts of the image. Since different muscles cooperate when performing different movements, changes within the images are usually not independent, but are coordinated together. The MACI method exploits these dependent coordination patterns naturally, and uses the whole array of stacked images when extracting its information. It was confirmed by speckle tracking that the MACI method can accurately identify the movement patterns that are present in ultrasound sequences, which is encouraging.

The MACI method can in fact also be used to find regions of interest, and not only using them. This is done by utilising the loading vectors, and reconstructing what we call *loading images *[25]. Such images give information of what areas where important for building for example a PCA model, and can thus be used to see which areas are active in different phases of a movement. However, this methodology has not yet been properly developed to be used in muscle tissue application. This is some of the future work that will be performed in order to improve the MACI method.

The MACI approach also allows comparative studies to be made between patients. Different groups of patients, for example healthy and control, could be modelled individually or together and compared for similarities and differences. That has not been done in this study, however, but is one of the aims of our future studies.

The MACI method examines image sequences inductively, without being told what to look for. Instead, a data driven method extracts the information that is the most important for describing a particular sequence. It is thus not the analyst who decides what parts of the image contribute the most information, but the method that does so. This unbiased data exploration approach therefore has great potential in clinical situations.

## Conclusions

The MACI method was able to correctly capture each passive reference movement and to describe the different phases of the ROM. It also captured the tissue dynamics during the different phases, partly in terms of the directions of the frames in the score plot, and partly in terms of distance relationships between images during the various movement phases. Also, both standardised and functional movements could be analysed with the MACI method.

To conclude, all sequences were modelled well with the MACI method and the different phases of the movements (turning phases, dorsal and plantar flexions) could all be identified and correlated in the three types of ankle movements. The same kind of pattern also arose when repeated flexions were compared; the repeatability provided by the MACI method is thus very encouraging.

Applying multivariate techniques based on whole image sequences is thus a powerful way to study variations and relationships between images in an ultrasound image sequence.

We argue that a multitude of applications are possible. The functionality of healthy muscles, injured muscles or training could be a focus. But also neuro-motor related diseases, such as multiple sclerosis, could be a possible area of application. Future studies will be made that will give more insight into what application areas are possible.

## Competing interests

Financial support for TL has been provided from MKS Umetrics AB, Umeå, Sweden. JT also serves on the scientific advisory board for MKS Umetrics AB, Umeå, Sweden.

## Authors' contributions

MP and TL contributed equally to the manuscript and the data analysis in this study. SV and HS laid the groundwork for the analysis in this study. MP and AA generated the data. JT and MP designed and coordinated this study. All authors read and approved the final manuscript.

## Pre-publication history

The pre-publication history for this paper can be accessed here:

http://www.biomedcentral.com/1471-2342/10/9/prepub

## Supplementary Material

Additional file 1 **Supplemental material**. This file contains more thorough expositions of some topics of the manuscript. The topics explained are: greyscale B-scan ultrasound, more on how it works and what anatomical landmarks are visible; speckle tracking, more on how it works; and information on how to interpret the speckle tracking displacement plots. Click here for file
