# Supplementary material for: Modelling human musculoskeletal functional movements using ultrasound imaging
Source: BMC Med Imaging. 2010 May 21;10:9. doi: 10.1186/1471-2342-10-9 (PMC2887375; doi:10.1186/1471-2342-10-9)
Supplement: Additional file 1 — Supplemental material. This file contains more thorough expositions of some topics of the manuscript. The topics explained are: greyscale B-scan ultrasound, more on how it works and what anatomical landmarks are visible; speckle tracking, more on how it works; and information on how to interpret the speckle tracking displacement plots. [file 1471-2342-10-9-S1.PDF]

Supplemental material for:

Modelling human musculoskeletal functional movements using ultrasound imaging

Michael Peolsson[3,*], Tommy Löfstedt[1,2,*], Susanna Vogt[1,2], Hans Stenlund[1,2], Anton Arndt[4,5] and Johan Trygg[1,2,†]

[1]Computational Life Science Cluster (CLiC), Umeå University, Umeå, Sweden
[2]Department of Chemistry, Umeå University, Umeå, Sweden
[3]School of Technology and Health, Royal Institute of Technology, Stockholm, Sweden
[4]Department of Clinical Science, Intervention and Technology, Division of Orthopeadic Surgery, Karolinska University Hospital, Huddinge, Stockholm, Sweden
[5]Swedish School of Sport and Health Sciences (GIH), Stockholm, Sweden

[*]These authors made equal contributions

[†]Corresponding author. Phone: +46907866917. Fax: +46907867655. E-mail: Johan.Trygg@chem.umu.se

1 Greyscale B-Scan Ultrasound

Ultrasound images are generated by transmitting longitudinal waves that are reflected during propagation in the tissues. The B-scan tissues are visualised as mismatches between transmitted and received signals as a function of travelling through media with different acoustic impedances. When the ultrasound wave enters a medium with a higher acoustic impedance the reflected echo will result in a bright area in the greyscale image and vice versa.

The main anatomical landmarks of the skeletal muscle provided by greyscale B-scan in this study are: the skin layers, the bone, the fascias, the muscle fascicles, the tendon and often also the bones. These structures are seen as brighter areas in the ultrasound images.

Three fascias are of importance: endo-, peri- and epimysium. The muscle fibers are covered by a thin sheet called *endomysium.* Fascicles, in turn, contain the muscle fibers and are sheltered by the *perimysium.* The anatomical muscle, enclosing the fascicles, is covered by the *epimysium.* The muscle fibers connect to a central tendon running through or along parts of a muscle and the tendon is attached in both ends to a bone structure. When using a high resolution linear 12 MHz probe the epi- and perimysium can be visualised, and tendons, but not the endomysium.

Anatomically, muscles are often located close to each other. Therefore, the whole ultrasound image will carry information about the coordination of muscle activities. Muscles change their shape during muscle performances, i.e. different closely related muscles may expand or shrink its volume during different phases of a movement. This implicates that capturing muscle dynamics is part of a time-place-shape related issue. As a consequence the wavelet based image analysing technique is appropriate.

2 Speckle Tracking

The acoustic patterns in an ultrasound signal change when the muscle being scanned moves and the objective of speckle tracking is to follow these patterns, the speckles, frame-by-frame in the loop.

Speckle tracking methods have been used within cardiac application fields to provide an angular independent tissue velocity tracking tool in two dimensions [1, 2, 3]. Speckle tracking has been validated in phantom and in vivo by sonomicrometry crystals [4]. A commercial software package (Echopac, GE Healthcare, Horten, Norway) was used together with the

ultrasonic system Vivid 7 (GE Healthcare, Horten, Norway) as a reference technique in the case examples in this study.

The principle idea of speckle tracking is that different parts of an image (e.g. of muscle tissue) have different speckle patterns. These speckle patterns remain relatively stable over time and can therefore be followed in a sequence of images. Thus, tissue movements and deformations can be captured in real-time during activity.

The first step in speckle tracking is to specify a rectangular region of interest (ROI) in a particular frame. The method finds the corresponding region in the next frame that is most similar to the selected region, by some criterion. I.e. the objective is to find the values of Δx and Δy that minimise

$$\varepsilon = \sum_y \sum_x \left[I(x, y, t) - I(x + \Delta x, y + \Delta y, t + 1)\right]^2 w(x, y), \qquad (1)$$

where I is the image intensity, x and y are the pixel coordinates at time, or frame, t and w is a weighting function, which can be 1 in the simplest case.

3 Understanding the Movements Captured by Speckle Tracking

The images in Figure 1 illustrate the reference movement. Four frames, out of approximately 100 frames, are extracted to illustrate the identification of the ankle positions. These are: Start point at rest, maximal dorsal flexion, maximal plantar flexion and end point at rest, respectively. The original greyscale loop is presented in the upper left part of each of the four images and a region of interest (ROI) is specified within the Achilles tendon for reference speckle tracking. The right part of each image presents the displacement, as a function of time, for each of the coloured segments of the ROI. The bottom left part of the images is a parametric image presenting the result in an alternative way—it is not used in the analysis.

It was clearly visible when watching the sequence that the ROI is moving in accordance with the actual movement being performed in a right-to-left direction along with the muscles.

Figure 1(a) presents the foot at rest, in a neutral position with 0 degrees flexion, and the ROI is inserted in a vertical position. Figure 1(b) presents the Achilles tendon segment at the turning point with 20 degrees of dorsal flexion. Figure 1(c) presents the turning point with 20 degrees plantar flexion. Finally, Figure 1(d) shows the Achilles tissue position when returned to the neutral ankle position at rest.

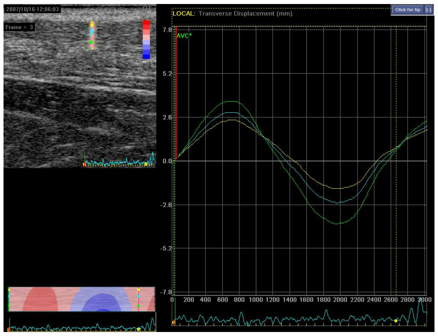

(a) Start point

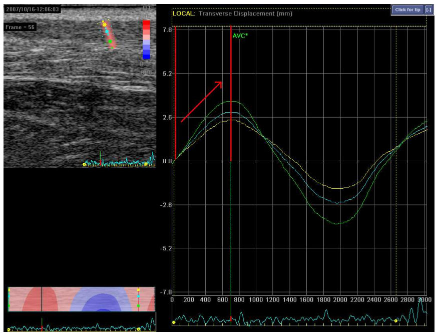

(b) Dorsal flexion, end/turn point

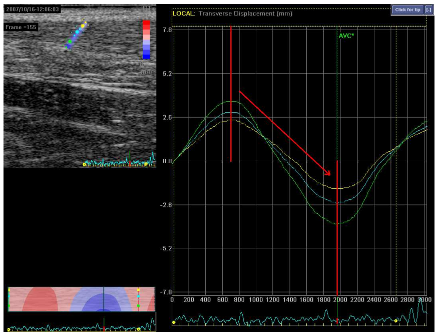

(c) Plantar flexion, end/turn point

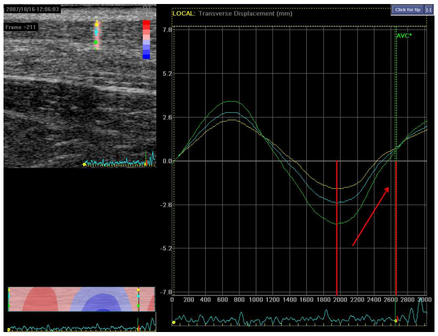

(d) End point

Figure 1: Reference images identifying the dorsal and plantar flexions. This is a confirmation of the direction of each movement. The right part of each image presents the displacement of the tendon as a function of time and range of motion.

References

[1] K. Kaluzynski, Chen Xunchang, Stanislav Y. Emelianov, Andrei R. Skovoroda, and Matthew O'Donnell. Strain rate imaging using two-dimensional speckle tracking. *IEEE Transactions on Ultrasonics, Ferroelectrics, and Frequency control*, 48(4):1111–1123, 2001.

[2] Abd El-Monem El-Sharkawy, Khaled Z. Abd-Elmoniem, Abou-Bakr M. Youssef, and Yasser M. Kadah. Improved ultrasound speckle motion tracking using nonlinear diffusion filtering. volume 4325, pages 453–461. SPIE, 2001.

[3] Marina Leitman, Peter Lysyansky, Stanislav Sidenko, Vladimir Shir, Eli Peleg, Michal Binenbaum, Edo Kaluski, Ricardo Krakover, and Zvi Vered. Two dimensional strain–a novel software for real-time quantitative echocardiographic assessment of myocardial function. *Journal of the American Society of Echocardiography*, 17(10):1021–1029, 2004.

[4] Josef Korinek, Jianwen Wang, Partho P. Sengupta, Chinami Miyazaki, Jesper Kjaergaard, Eileen McMahon, Theodore P. Abraham, and Marek Belohlavek. Two-dimensional strain–a doppler-independent ultrasound method for quantitation of regional deformation: Validation in vitro and in vivo. *Journal of the American Society of Echocardiography*, 18(12):1247–1253, 2005.
